# Lithium and preventing chemotherapy-induced peripheral neuropathy in breast cancer patients: a placebo-controlled randomized clinical trial

**DOI:** 10.1186/s13063-021-05800-w

**Published:** 2021-11-24

**Authors:** S. Najafi, Z. Heidarali, M. Rajabi, Z. Omidi, F. Zayeri, M. Salehi, S. Haghighat

**Affiliations:** 1 Breast Cancer Research Center, Motamed Cancer Institute, ACECR, Tehran, Iran; 2 Department of Clinical Pharmacy, Faculty of Pharmacy, Tehran Medical Sciences, Islamic Azad University, Tehran, Iran; 3 Department of Clinical Pharmacy, University Hospitals of North Midlands NHS Trust, Stoke-on-Trent, ST46QG UK; 4 Proteomics Research Center and Department of Biostatistics, School of Allied Medical Sciences, Shahid Beheshti University of Medical Sciences, Tehran, Iran; 5 Department of Biostatistics, School of Public Health, Iran University of Medical Sciences, Tehran, Iran

**Keywords:** Chemotherapy, Prevention, Peripheral neuropathy, Lithium, Breast cancer

## Abstract

**Background:**

Chemotherapy-induced peripheral neuropathy (CIPN) is a common adverse effect of some chemotherapy regimens. Lithium has been suggested for CIPN in some animal studies. We aimed to study lithium’s preventive effect on CIPN in breast cancer patients treated with taxanes and platinum-based medications.

**Method:**

A double-blind placebo-controlled randomized clinical trial (RCT) was implemented on 36 breast cancer patients in two equal-size groups by block randomization. Participants in both groups consumed daily tablets, either placebo or lithium (300 mg), for 5 days in each course of chemotherapy. The tablets were prescribed 1 day before the start of chemotherapy. The electromyography (EMG) and nerve-conduction-velocity (NCV) tests were achieved before the first chemotherapy, 3 and 9 months after the treatment. The changes and signs or symptoms of CIPN, such as numbness, tingling, freezing, sensitivity to touch, muscle weakness, fibrillation, and knee and elbow reflex disorders, were recorded by examination. The trend of outcome changes was compared between two groups during the 9 months of study.

**Results:**

In both groups, neurologic signs and symptoms were exacerbated during the first 3 months and improved up to the ninth month of study. Results showed significant changes of all EMG-NCV variables during the 9 months of research in each group (*P* < 0.001), but the interaction of time and group effect was not significant in none of those indices. All symptoms changed significantly over the study time (*P* < 0.001) without significant statistical difference between the two groups (*P*=0.352). No side effect was found during the study.

**Conclusion:**

The study showed that 300 mg lithium prescription once daily for 5 days might not effectively prevent CIPN in breast cancer patients. Evaluation of lithium effect on CIPN on different cancers in future studies is suggested.

**Trial registration:**

Iranian Registry of Clinical Trials IRCT20160813029327N10.  Registration date: May 16, 2018.

## Introduction

Chemotherapy-induced peripheral neuropathy (CIPN) is one of the prevalent adverse effects of neurotoxic chemotherapy in cancer patients [1]. The appearance of CIPN is often dose-dependent, and the severity of this complication increases with the increasing accumulation of neurotoxic substances. Most chemotherapeutic medicines causing CIPN are platin compounds, vinca alkaloids, taxanes, Bzomib, and Thalidomide. They are predominantly used in solid and hematological malignancies such as breast cancer and multiple myeloma [2, 3]. As a result, the adverse events associated with CIPN could lead to changes in the quality of life and independent performance of daily living activities [4]. After the initiation of chemotherapy, CIPN develops within weeks or months, and it may even last from months to years after the completion of chemotherapy.

Patients with CIPN typically suffer from paresthesia (tingling, numbness), pain, and muscle weakness resulting in a significantly diminished quality of life [5]. This peripheral nerve toxicity commonly is bilateral and symmetric and is often referred to as a “stocking-glove” neuropathy. Moreover, evidence has shown that one-third of CIPN cases could become permanent [6].

Numerous studies have shown that chemotherapeutic agents mentioned above cause CIPN via different mechanisms such as disrupted microtubule-mediated axonal transport, axonal degeneration, direct damage to the dorsal root ganglion (DRG), and mitochondrial dysfunction [4, 7].

Several compounds have been studied to prevent CIPN, including intravenous calcium and magnesium, vitamin E, amifostine, glutathione, glutamine, acetyl-L-carnitine, N-acetyl-cysteine, antiepileptic medicines, and others. The studies were limited by small sample sizes and lacked placebo-controlled randomized designs [8–13]. For instance, according to a placebo-controlled clinical trial in Iran (2012), the incidence of CIPN in patients given omega-3 fatty acids was 30% less than in the placebo group [14, 15].

Lithium has neuroprotective and anti-pain effects. It is a well-known drug that is used as a mood stabilizer medicine among bipolar patients. Lithium directly inhibits two signal transmission paths. The first path is inhibition of inositol signaling by discharging intracellular inositol, and the second path, inhibiting GSK3-ß, multitask protein kinas. Previous studies have shown that GSK3B in the neuroinflammation process is highly concerned. Lithium also indirectly has some impressions on neurotransmitters releasing [16]. It induces neuroprotective effects in central nervous system and improves sensory neuropathy related to the chemotherapy drugs [17–19].

A few animal studies have examined the effectiveness of lithium for the prevention of CIPN. These studies have shown that lithium could be useful in preventing CIPN [17–19]. Moreover, one case report has demonstrated that lithium could decrease the chances of developing CIPN [20]. So, in this research, we intended to discuss the effect of lithium in the prevention of CIPN specifically.

Overall, chemotherapy-induced peripheral neuropathy is a common side effect of some chemotherapeutic agents that impresses patients’ quality of life and daily activities. This adverse effect is either partially or entirely reversible [21]. There have been a few animal trials looking at lithium as a possible neuroprotective effect in neuropathy, but there was no available human clinical trial in this regard. So, this clinical trial was developed to evaluate the efficacy and safety of 300 mg lithium tablets for CIPN prevention in breast cancer patients undergoing chemotherapy.

## Methods

### Trial design

This study was a double-blind, parallel randomized placebo-controlled clinical trial to assess lithium’s efficacy and safety for CIPN prevention in breast cancer patients undergoing chemotherapy. The method of assigning individuals to groups was randomized blocks of four. Patients were randomly assigned to the drug (*n*=18) or the placebo (*n*=19) groups by a ratio of 1:1. The study was approved by the Ethics Committee of the Islamic Azad University of Pharmaceutical Sciences (IAUPS), Tehran, Iran (No: 374).

### Participants

The inclusion criteria were having a breast cancer diagnosis, aged between 18 and 60 years old, undergoing chemotherapy, and agreement for participation by signing informed consent. The chemotherapy regimen consisted of four courses of Adriamycin-cyclophosphamide with four courses of taxanes-based medicines (AC4*T4). The exclusion criteria consisted of lack of interest to participate; comorbidities such as diabetes, cardiovascular disorders, renal disorders, bipolar, and thyroid disorders; the presence of peripheral nerve damage due to another illness and/or medication; pregnant or breastfeeding status; planning to become pregnant; opium addiction; lithium intoxication; and manifesting dangerous complications.

### Intervention

Interested eligible patients were enrolled randomly by the blinded researcher from November 2017 to June 2019 at the Breast Clinic of Motamed Cancer Institute in Tehran, Iran. After obtaining written consent, patients were randomly assigned to one of the drug or placebo groups.

The placebo group patients were given one placebo tablet 1 day before initiating each chemotherapy cycle and continuing 4 days (5 days-one tablets per day). The placebo tablets were manufactured at the Industrial Laboratory of Islamic Azad University of Pharmaceutical Sciences Branch, Tehran, Iran. The placebo tablets’ main ingredients included starch, lactose, Avicel (microcrystalline cellulose), and PVP (polyvinyl pyrrolidone).

The drug group patients were given 300 mg lithium carbonate tablets, production of Tehran Darou Pharmaceutical Co. Tehran, Iran, for five consecutive days, starting 1 day before each chemotherapy cycle. Lithium is a well-known drug that is used as a mood stabilizer medicine among bipolar patients. We applied an intervention with a low dose of lithium carbonate due to the lack of human studies, ethical issues, and lithium interference with the Adriamycin-cyclophosphamide regimen. Carbonate salt is the only salt of lithium that exists in the Iranian pharmaceutical market. It is completely absorbed within 6–8 h, with plasma peak levels in 30 min to 2 h. Lithium is distributed in total body water and intracellular compartment slowly. Its initial volume of distribution is 0.5 L/kg, then rising to 0.7–0.9 L/kg. It has some sequestration in bone without protein binding. Lithium excretion is in the urine with a plasma half-life of about 20 h. According to the Nice guideline, lithium carbonate dosage is different in patients with a bodyweight of < 50 and > 50 kg. It can be prescribed up to 400 mg in a bodyweight of > 50 kg. Since most participants were > 50 kg, a daily dose of 300 mg was used to prevent side effects [22]. The placebo tablets and lithium carbonate tablets had a round, white, and striped appearance and thickness.

**Fig. 1:**
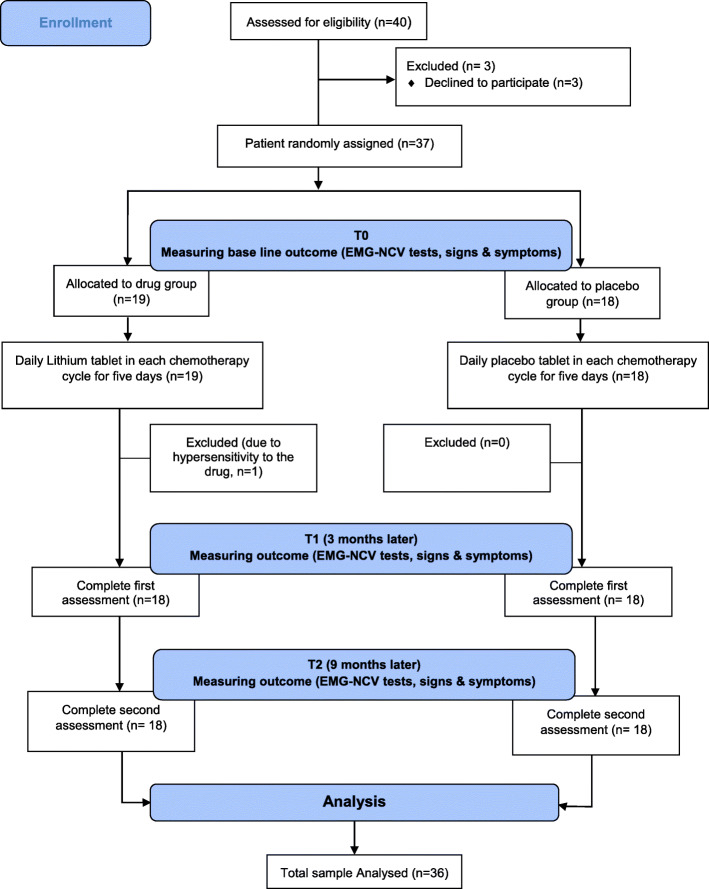
Consort diagram: recruitment and eligibility screening, randomization, follow-up, and analysis

At the start of the study, participants’ demographic and clinical characteristics were recorded in a checklist. EMG-NCV tests in four limbs and neurologic signs and symptoms were measured before starting chemotherapy, 3 and 9 months after it. They were compared and evaluated in each group and between the two groups. Figure 1 shows a summary of the study design.

### Outcome

1. Signs and symptoms: these included numbness, tingling, freezing, sensitivity to touch, muscle weakness, and knee and elbow reflexes and were measured before chemotherapy, 3 and 9 months after starting chemotherapy by a blinded oncologist through examination.
2. EMG-NCV: these tests were taken by a blinded neurologist before chemotherapy, 3 and 9 months after starting chemotherapy.

### Instrument

Demographic and clinical characteristics such as age, reproductive status, stage of disease, lymph node number, tumor size, and hormone therapy were recorded by a checklist. During the 9 months of study, all patients’ signs and symptoms were recorded in a clinical registry form by the physician’s examination. The data of EMG-NCV indices were extracted from the neurologist’s report.

### Sample size

The required sample size was calculated according to an animal study. In that study, sensory-motor activity was evaluated by the rotarod instrument. The length of time on the rotarod for the paclitaxel-saline-treated and paclitaxel-lithium-treated mice dropped to 67 + 6% and 5 + 9%, respectively, compared to vehicle-saline-treated mice. Considering the power of 0.8, significant level *α*=0.05, and a 10% dropout rate, the final sample size was estimated to be twenty patients in each group. So, forty patients were included in the study.

### Randomization

In this study, a random block of four was provided in closed envelopes by a statistician. Letters A and B were considered intervention (chemotherapy + lithium) and placebo (chemotherapy + placebo) groups. The blocks consisted of equal numbers of A and B drugs with a random arrangement. Each eligible patient was enrolled in one of the two groups randomly through the available sampling method by a trained researcher. All the information about the type of intervention was confidential, and patients and investigators were not aware of the allocated groups. The tablets and the packs containing them were identical in appearance, size, and color to achieve this goal.

### Statistical analysis

Results were analyzed using the Statistical Package for Social Sciences (SPSS) version 22. Considering the small sample size (less than 30 patients in each group), we used nonparametric tests despite the normal distribution of variables to increase this study’s power.

The chi-square test evaluated the frequency difference of the clinical and demographical variables between groups. Changes of EMG_NCV over the study time were assessed by the Friedman test. The generalized estimating equations (GEE) tests were performed to evaluate three-time spans’ interaction effect (before starting chemotherapy, 3 and 9 months after it) and two groups on the EMG_NCV changes. The interaction of time and group effect on symptom score was studied by GEE, too.

## Results

Thirty-seven subjects were divided into lithium (*n*=19) and placebo (*n*=18) groups randomly. Yet, one patient was excluded from the lithium group due to hypersensitivity to the drug. Some demographic and clinical characteristics are summarized in Table 1. Most patients were aged 27 to 60 years, and the mean age was 45 years. More than 50% of patients were in menopause. There was no significant difference in age distribution and reproductive status between the two groups (*P*=0.515, *P*=0.735, respectively). More than 50% of patients were stage II breast cancer in terms of clinical features, 77.8% of patients had more than one affected lymph node, and 11.1% had a tumor size of more than 5 cm.

**Table 1:** Demographic and clinical characteristics of each group

Variable	Drug *N* (%)	Placebo *N* (%)	*P* value
**Menopausal statement**			0.735
Pre-menopause	8 (44.4)	7 (38.9)	
Menopause	10 (55.6)	11 (61.1)	
**Stage of disease**			0.728
1	1 (5.6)	1 (5.6)	
2	10 (55.6)	11 (61.1)	
3	7 (38.9)	6 (33.3)	
**Affected lymph nodes**			1
0	4 (22.2)	4 (22.2)	
≥1	14 (77.8)	14 (77.8)	
**Tumor size (cm)**			0.566
< 2	5 (27.8)	6 (33.3)	
≥ 2–5	10 (55.6)	11 (61.1)	
≥ 5	3 (16.7)	1 (5.6)	
**Hormone therapy**			0.682
Not received	3 (16.7)	3 (16.7)	
Tamoxifen	3 (16.7)	2 (11.1)	
Letrozol	6 (33.3)	5 (27.8)	
Tamoxifen and letrozol	0 (0)	2 (11.1)	
Tamoxifen and Decapeptyl	6 (3.3)	6 (33.3)	
**Frequency of Taxol/Taxotere injection**			1
4	17 (94.4)	17 (94.4)	
> 4	1 (5.6)	1 (5.6)	

All subjects received AC4*T4 chemotherapy regimen without Herceptin administration. Concurrent use of tamoxifen and Decapeptyl was prescribed in 33.3% of patients. None of the patients had a history of diabetes or renal or heart failure. All patients were monitored by phone, and their adherence rate to drug/placebo use was 100%. There was no significant difference between the two groups regarding clinical characteristics, which approves participants’ random assignment to the two groups.

EMG-NCV characteristics of subjects in the two groups are shown in Table 2. Assessment of outcome changes during the study period by the Friedman test showed that time significantly impacted all EMG-NCV variables (*P*< 0.001).

**Table 2:** The distribution of EMG-NCV (Median and IQR) in each group of study

Variable	Group	Start time Median (IQR)	3rd month Median (IQR)	9th month Median (IQR)	***P***
**Ulnar NCV**	Drug	65 (9)	42.5 (15.3)	55 (17)	< 0.001
	Placebo	68.5 (5)	40 (17.8)	52 (18.5)	< 0.001
**Ulnar Amp**	Drug	6.1 (1)	3 (2)	5.4 (1.2)	< 0.001
	Placebo	6.4 (1)	4 (1.5)	5 (2)	< 0.001
**Median NCV**	Drug	60 (18.5)	40 (18.5)	50 (14.5)	< 0.001
	Placebo	60.5 (10)	39 (17.5)	49.5 (18.3)	< 0.001
**Median Amp**	Drug	5.2 (1.4)	2.9 (2.1)	4.1 (2)	< 0.001
	Placebo	5 (1.6)	2.3 (2)	4.3 (2.3)	< 0.001
**Tibial NCV**	Drug	53.5 (10)	36.5 (16.5)	45.5 (8.3)	< 0.001
	Placebo	55 (7.5)	34.5 (19.3)	47.5 (13.3)	< 0.001
**Tibial Amp**	Drug	3 (0.1)	1.9 (1.3)	2.4 (0.8)	< 0.001
	Placebo	3 (0.2)	1.8 (1.2)	2.5 (1)	< 0.001
**Proneal NCV**	Drug	54 (11.5)	35 (13.3)	42.5 (12.5)	< 0.001
	Placebo	54.5 (10.8)	32 (11.5)	43.5 (15.9)	< 0.001
**Proneal Amp**	Drug	3 (0.3)	1.9 (1.3)	2.3 (0.7)	< 0.001
	Placebo	3 (0.1)	1.9 (0.9)	2.1 (1)	< 0.001

*NCV* nerve conduction velocity, *Amp* amplitude

The frequency of signs and symptoms over time between the two groups has been presented in Table 3. At the beginning of chemotherapy, no patient had fibrillation. Three months later, fibrillation was observed in most patients of both groups. Lack of fibrillation in the drug group was 5% more than the placebo group, and severe fibrillation (3+) in the drug group was 5% less than the placebo group. In the 9th month, severe fibrillation was observed in none of the patients, but the drug group’s lack of fibrillation was about 10% more than the placebo group.

**Table 3:** Frequency of symptoms between two groups in three measurement status

Variable	Group	Start time ***N*** (%)	3rd month ***N*** (%)	9th month ***N*** (%)
Fibrillation	Drug			
	0	18 (100)	7 (38.9)	16 (88.9)
	1^+^	0 (0)	2 (11.1)	0 (0)
	2^+^	0 (0)	7 (38.9)	2 (11.1)
	3^+^	0 (0)	7 (11.1)	0 (0)
	Placebo			
	0	18 (100)	6 (33.3)	14 (77.8)
	1^+^	0 (0)	3 (16.7)	2 (11.1)
	2^+^	0 (0)	6 (33.3)	2 (11.1)
	3^+^	0 (0)	3 (16.7)	0 (0)
Numbness	Drug	0 (0)	12 (66.7)	2 (11.1)
	Placebo	0 (0)	12 (66.7)	4 (22.2)
Tingling	Drug	0 (0)	18 (100)	11 (61.1)
	Placebo	0 (0)	18 (100)	12 (66.7)
Freezing	Drug	0 (0)	12 (66.7)	10 (55.6)
	Placebo	0 (0)	12 (66.7)	10 (55.6)
Sensitivity to touch	Drug	0 (0)	2 (11.1)	0 (0)
	Placebo	0 (0)	6 (33.3)	0 (0)
Muscle weakness	Drug	0 (0)	1 (5.6)	0 (0)
	Placebo	0 (0)	1 (5.6)	0 (0)
Elbow reflex	Drug	18 (100)	17 (94.4)	17 (94.4)
	Placebo	18 (100)	14 (77.8)	18 (100)
Knee reflex	Drug	18 (100)	16 (88.9)	18 (100)
	Placebo	18 (100)	15 (83.3)	17 (94.4)

At the beginning of chemotherapy, no patients had signs and symptoms of numbness, tingling, freezing, sensitivity to touch, muscle weakness, or elbow and knee reflexes disorder. Three months later, numbness, tingling, and freezing were the same in the studied groups. Sensitivity to touch in the placebo group was 22% more than the drug group, and muscle weakness was observed similarly in the two groups. Elbow and knee reflex disorders were 17% and 5% more than the drug group in the placebo group, respectively. In the 9th month, numbness and tingling incidence in the placebo group decreased to 10% and 5% compared to the drug group. The frequency of freezing was completely similar between the groups during the study.

The interaction of group and time on EMG_NCV indices is shown in Table 4. To increase study power and assess the effect of contextual variables in frequent measurements, a generalized estimating equation (GEE) test was used. According to the results, the interaction of time and group effect was insignificant in none of the EMG_NCV indices.

**Table 4:** GEE analysis of the interaction of group and time on EMG_NCV indices

Variable		***B***	Std. error	Wald chi-square	***P*** value
**Ulnar NCV**	G2/1 * T9/0	3.222	3.5649	.817	.366
	G2/1 * T3/0	5.000	3.3491	2.229	.135
	G2/1 * T9/3	− 1.778	1.4577	1.487	.223
**Ulnar Amp**	G2/1 * T9/0	.333	.3128	1.136	.287
	G2/1 * T3/0	− .117	.4228	.076	.783
	G2/1 * T9/3	.450	.3670	1.503	.220
**Median NCV**	G2/1 * T9/0	.500	2.6094	.037	.848
	G2/1 * T3/0	2.889	3.2224	.804	.370
	G2/1 * T9/3	− 2.389	1.9513	1.499	.221
**Median Amp**	G2/1 * T9/0	− .222	.2185	1.034	.309
	G2/1 * T3/0	.100	.3142	.101	.750
	G2/1 * T9/3	− .322	.3191	1.019	.313
**Tibial NCV**	G2/1 * T9/0	.056	2.6936	.000	.984
	G2/1 * T3/0	1.889	3.3171	.324	.569
	G2/1 * T9/3	− 1.833	2.3878	.589	.443
**Tibial Amp**	G2/1 * T9/0	− .244	.1706	2.052	.152
	G2/1 * T3/0	− .150	.2017	.553	.457
	G2/1 * T9/3	− .094	.1605	.346	.556
**Proneal NCV**	G2/1 * T9/0	.083	2.7807	.001	.976
	G2/1 * T3/0	3.056	3.7217	.674	.412
	G2/1 * T9/3	− 2.972	2.1796	1.860	.173
**Proneal Amp**	G2/1 * T9/0	− .067	.2034	.107	.743
	G2/1 * T3/0	.022	.2310	.009	.923
	G2/1 * T9/3	− .089	.1557	.326	.568
**Symptoms**	Group 2/1	.138	.19	.54	.386
	time	.061	.01	55.99	<.001
	G2*T9/0	− .003	.01	.05	.352

G2/1: Group 2 compared to group 1

T9/0: 9th months compared to start time

T3/0: 3rd months compared to start time

T9/3: 9th months compared to 3rd months

*NCV* nerve conduction velocity, *Amp* amplitude

**Fig. 2:**
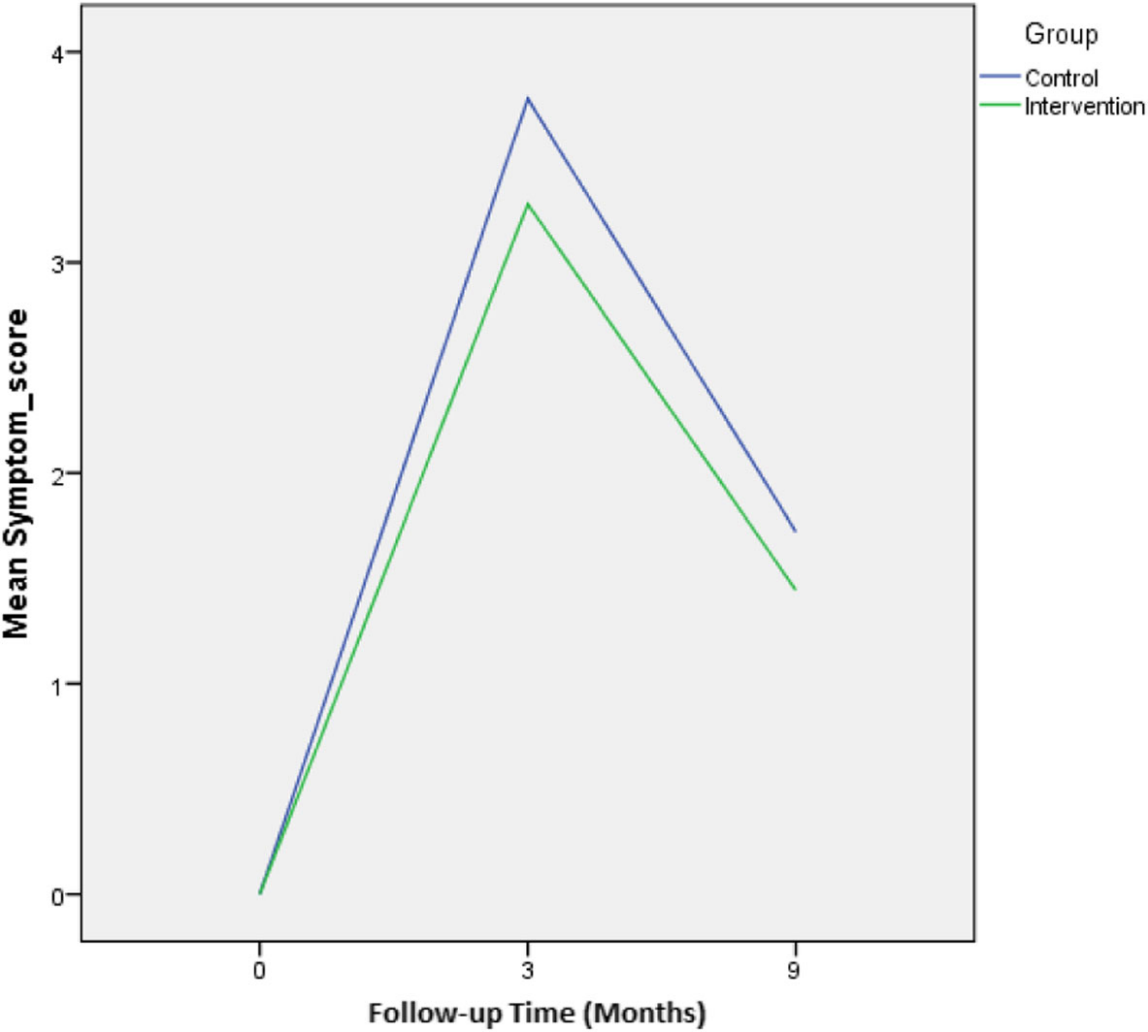
Comparing changes of symptoms two groups

A new index has been created by computing eight symptoms together due to the high degree of variance related to symptom variables. These effects have been presented in the last row of Table 4. Although symptoms had changed significantly over the study time (*P*< 0.001), its interaction with group effect was not significant during the 9 months of follow-up (*P*=0.352). Comparing changes of symptoms between the two groups have been demonstrated in Fig. 2.

## Discussion

Despite encouraging animal studies, this double-blind, placebo-controlled RCT showed that lithium administration was not advantageous or beneficial for the prevention of CIPN. As a preventive measure, lithium did not significantly affect peripheral neuropathy following chemotherapy, according to the results.

To date, different studies have evaluated pharmacological and non-pharmacological interventions in preventing CIPN. Yet, most had a small sample size with conflicting results. Only a small number of RCTs have evaluated natural products, and one has evaluated electro-acupuncture.; needless to say, thorough studies are required to confirm their efficacy [23]. Therefore, in this study, an attempt was made to use a logical sample size.

As a multifunctional drug, lithium directly inhibits two signal transmission paths and indirectly impressions on neurotransmitters releasing [16]. So, it can be concluded that it has neuroprotective effects through several mechanisms. The delay between the start of treatment and the emergence of clinical outcomes suggests that lithium’s effect is mainly through the cellular messengers and eventually a change in gene expression and neuroprotective effects of lithium. One case reports clinical trial has shown that lithium could decrease the chances of developing CIPN. In this study, 114 patients with different cancers were going through chemotherapy. Four of them took lithium or valproic acid for several years due to a history of bipolar disorder or schizophrenia. None of these four patients showed any signs or symptoms of worsening or developing peripheral neuropathy. Results showed that treatment with lithium or valproic acid concurrently with chemotherapy was associated with a decreased incidence of developing CIPN and, therefore, increased quality of life during and after chemotherapy. This study has claimed that lithium or valproic acid could have a neuroprotective effect through inhibiting neuronal calcium sensor 1(NCS1) actions [20].

Moreover, a few animal studies have examined the effectiveness of lithium for the prevention of CIPN. These studies have shown that lithium could be useful in preventing CIPN [17–19]. One of them, published in 2012, has proved that a single dose of lithium injected into rats before using Paclitaxel could prevent CIPN. This article claims that lithium’s possible mechanism to avoid CIPN is inhibiting calcium signaling [19]. The results of another study showed that lithium could prevent sensory and motor neuropathy symptoms from vincristine administration via inhibition of glycogen synthase kinase 3beta (GSK3B) [17]. A study that assessed inhibition of glycogen synthase kinase 3beta activity with lithium prevents and attenuates paclitaxel-induced neuropathy pain showed that lithium could prevent neuropathic pain from Taxol consumption through inhibiting GSK3B [18]. Although lithium has neuroprotective and anti-pain effects, this research specifically focused on its impact on the prevention of CIPN. This study cannot assess many signal transmission paths.

In the current research, chemotherapeutic agents and lithium were administered simultaneously. Initially, the interaction of chemotherapy medicines with lithium was investigated. Chemotherapy medicines used by patients had no intervention with lithium, and they did not disturb the treatment process. Also, we considered inclusion criteria strictly. Moreover, to prevent toxicity and adverse reaction, the minimum daily dose used in bipolar, which is 300 mg, was considered in this study. Fortunately, prescribing a safe dose of the drug, no side effects were observed during the study. It is suggested that higher lithium’s dose and the more number of days consumption are studied in future researches

In our study, the results of EMG-NCV tests showed that all signs and symptoms, fibrillation, and reflexes were similar between the two groups at the beginning of the chemotherapy. After 3 months of chemotherapy, the lack of fibrillation, sensitivity to touch, elbow reflex, and knee reflex disorder in the drug group were observed 5%, 22%, 17%, and 5% more than the placebo group. In the ninth month of study, the drug group’s lack of fibrillation was about 10% more than the placebo group. At the end of the study, the placebo group claimed numbness and tingling symptoms 10% and 5% more than the drug group. Overall, the situation was better in the intervention group, but the difference between them was insignificant. One possible reason for this could be an insufficient time interval between starting lithium consumption and starting chemotherapy. Considering lithium’s pharmacodynamics and pharmacokinetic profile, it seems that administration of lithium before chemotherapy may cause more effective blood concentration. Designing a clinical trial with different time intervals and doses may provide useful evidence of lithium’s role in CTIN prevention and improvement of patients’ quality of life.

## Conclusions

According to the results obtained from the current trial, although EMG-NCV variables and symptoms changed significantly over time, there was no significant difference between the placebo and the lithium group in developing CIPN. So, it is suggested that the effect of the other doses of lithium be evaluated in future studies.

## Data Availability

The datasets generated and analyzed during the current study are available through contact with the corresponding authors: haghighat@acecr.ac.ir, sha_haghighat@yahoo.com.

## Abbreviations

EMG: Electromyography
NCV: Nerve conduction velocity
CIPN: Chemotherapy-induced peripheral neuropathy
AC4*T4: Adriamycin-cyclophosphamide four courses with taxanes-based medicines four courses
